# The Emotional Universe of Nonbinary Parents: A Hermeneutic Study

**DOI:** 10.3390/healthcare13121467

**Published:** 2025-06-18

**Authors:** Victoria Emilia Souviron-Dixon, Pablo Martínez-Angulo, María del Rocío Jiménez-Mérida, Pedro E. Ventura-Puertos

**Affiliations:** 1 Instituto Maimónides de Investigación Biomédica de Córdoba (IMIBIC), Universidad de Córdoba (UCO), Hospital Universitario Reina Sofía (HURS), 14004 Córdoba, Spain; victoria.souviron29@gmail.com (V.E.S.-D.); n02jimem@uco.es (M.d.R.J.-M.); pventura@uco.es (P.E.V.-P.); 2 Department of Nursing, Pharmacology and Physiotherapy, Faculty of Medicine and Nursing, University of Córdoba (UCO), 14004 Córdoba, Spain; 3 Interdisciplinary Research Group in Discourse Analysis (HUM380), University of Córdoba (UCO), 14071 Córdoba, Spain

**Keywords:** sexual and gender minorities, parenting, gender diversity, gender binary, emotions

## Abstract

**Introduction**: Nonbinary individuals who do not identify as exclusively male or female often face unique emotional challenges due to societal cisheteronormativity and limited recognition of their identities. While existing research has primarily focused on anxiety, depression, and pathways to parenthood among nonbinary people, little attention has been paid to their comprehensive emotional experiences as parents. This study aims to explore the emotional universe of two nonbinary parents from Spain and the United States. Design: Hermeneutic study. **Materials and Methods**: We implemented purposive sampling, conducted semi-structured virtual interviews, and followed Ricoeur’s theory of interpretation for data analysis. We used the Universe of Emotions affective taxonomy as a starting category in this analysis. Our sample consisted of a 32-year-old white Spanish nurse (she/they/them), assigned female at birth and parent of two one-year-old toddlers, and a 34-year-old white North American physiotherapist (he/they/them) assigned female at birth and parent of a ten-year-old child. **Results**: Through its four themes (*A story of misunderstanding: “What are you, a combat helicopter?”; Clearly, you don’t fit*, so…; *But (a new) family is there; No monster here: I’m, at the core, a human being*), this study reveals the complex emotional journey experienced by two nonbinary parents. **Conclusions**: Central to this journey are three key emotions: strangeness, belonging, and acceptance. The participants describe an initial stage marked by body and social dysphoria, confusion, and rejection, followed by a transformative process in which parenthood becomes a catalyst for emotional and identity integration. This transition—from alienation to connection—reflects a broader movement from dehumanization to humanization, where the experience of parenting fosters emotional resilience, social recognition, and a renewed sense of self. Implications for the profession and/or patient care: Analyzing their emotions (both negative and positive ones), we obtained robust insights into these parents’ personal and social contexts. Therefore, we can facilitate understanding of the emotional complexity of nonbinary parents by the trans and cisgender communities. Through this understanding, nurses and the organizations they work for can improve their competence in their holistic care. Acceptance from nonbinary parents’ social contexts, of which nursing is a part, is a critical factor in their health and emotional wellbeing.

## 1. Introduction

The acronym LGTBIQ+ stands for those people who define themselves as lesbian, gay, trans, bisexual, intersex, queer, or any other identity that does not conform with cisnormativity and/or heteronormativity. This community includes *trans* and gender-diverse people and, within these, nonbinary people [1].

Individuals who identify as nonbinary do not see themselves as exclusively either male or female, but can identify as any of them completely, partially, or not at all, or a completely different gender identity outside of a strict male–female gender dichotomy. Nonbinary people may use other pronouns, such as the gender-neutral pronoun “they” or neopronouns (e.g., “ze”, “sie”, “xe”) [1]. There are nonbinary people who define themselves as nonbinary transmasculine people or nonbinary transfeminine people, depending on whether they identify more with a masculine or feminine identity or gender presentation/expression, while still preserving the nonbinary essence of their gender identity [2]. Nonbinary people may choose to receive Gender Affirming Care (GAC) [1]. In the current clinical context, several studies have highlighted the absence of standardized, evidence-based guidelines for the comprehensive care of trans and nonbinary individuals, leading to inconsistent and sometimes iatrogenic practices [3]. This lack of clinical consensus adds to epistemological critiques warning of the risks associated with early medicalization and a singular ideological interpretation of gender identity [4].

In the international context, the dominant models defining gender identities that are applied increase reactions and expectations regarding people’s gender, making any gender identification, transition, and expression that breaks the binary categorization of gender difficult. Invisibilization, ignorance, and hateful reactions create specific conditions of vulnerability for nonbinary people globally and their access to health services and social opportunities [5]. Most describe this vulnerability through experiences such as avoidance of public spaces and services, fear of the consequences of their visibility, uncertainty in social interactions, isolation, loneliness, or feelings of distress. Therefore, it is not surprising that more than 90% of nonbinary people use “passing” strategies (gender concealment strategies) in at least one area of their lives, and more than half use passing in most of their environments [5].

The pressure to conform to a cisheteronormative society is associated with the emergence of gender dysphoria, which can be experienced by trans and gender-diverse people. Gender dysphoria can be identified as the psychological distress someone can suffer from a perceived incongruence between their gender identity and their physical characteristics associated with it. The experience of dysphoria is unique to every individual and may change in response to diverse physical, social, occupational, temporal, and psychological dynamics [2].

Recent evidence estimates that approximately one-fifth of trans people have been parents, and about one-third of them were nonbinary parents [6]. In spite of the emotional and psychological vulnerability described above, the positive experiences of parenthood may moderate or reduce some of the negative outcomes of stigma because their health outcomes are similar to cisgender parents [7].

Due to the normalization of transphobic attitudes in different sectors of society, trans people face many obstacles before becoming parents [8]. The parent’s gender transition, once achieved, often results in family resilience so that the family, including the children, embrace gender diversity references and tend to be more open-minded with marginalized groups in general. Many families with a trans parent report positive parent–child relationships [9].

As a consequence of the personal and social context of nonbinary people described above, they may suffer from vulnerability that impacts their health and wellbeing. When we speak of personal situations of vulnerability, we are implicitly referring to their or our emotional world, a world from which we construct a large part of our identity [10]. Emotions play a mediating role in the construction of identity within contexts of structural oppression, as highlighted by Minority Stress Theory [11], which explains how stigma, prejudice, and discrimination generate chronic stress that impacts the mental health of LGTBIQ+ individuals. Similarly, Queer Theory provides a critical lens on gender and sexuality norms, helping us to understand how emotions arise from the tension between lived identity and normative social expectations [12].

In an effort to “visualize the rich world of affective phenomena that we experience within ourselves […] as a strategy for a better understanding of them”, the authors of [13] created the Universe of Emotions taxonomy, comparing “the emotions to the cosmic universe as a metaphor” (p. 35). The author establishes its structure “around two great constellations”, one of positive emotions (the Galaxies of Joy, Love, and Happiness) and the other of negative ones (the Galaxies of Anger, Disgust, Fear, Anxiety, and Sadness). The Galaxy of Surprise is in a central position, the same as social emotions like Shame and Guilt [13].

Scientific research has focused mainly on anxiety and depression suffered by nonbinary people [14] and their different pathways to becoming parents [15]. But very little research has focused specifically on the integral emotional experiences of nonbinary parents. An effort is required to take a thorough approach to the emotional experiences of nonbinary parents, bearing in mind that through emotions, both negative and positive ones, we can obtain robust insights into these parents’ personal and social context. The present study aims to explore the emotional universe of two nonbinary parents from Spain and the United States.

## 2. Materials and Methods

### 2.1. Design

Our choice of a hermeneutical design is based on its ability to provide a deeper understanding of emotions [16], which we view as intrinsically linked to human experiences, albeit with our unique interpretation [17].

### 2.2. Participants and Settings

We employed a purposive intentional sampling strategy for participant selection, specifically targeting individuals who were experts on nonbinary parenting experiences. We also employed snowball sampling technique [18]. Our criterion for inclusion in the sample consisted of participants having to identify as nonbinary parents. In the same vein as Mayan [19], we understood that we had reached data saturation when we found in the data analysis results that we considered essential and novel about our research interest, without the pretension of saying everything, the only thing, nor the last thing about the phenomenon. Although our sample consisted of only two participants, this decision is grounded in a hermeneutic qualitative approach, where the concept of saturation is not determined by the number of cases but by the depth and richness of interpretive analysis. In intensive studies focused on understanding subjective experiences, even a single interview may suffice if it grants access to the cultural and emotional meanings of the phenomenon [20]. In our study, both participants were selected for their informational density and contextual diversity. The analysis was conducted through an iterative and reflexive process, allowing us to reach theoretical saturation regarding emerging categories and affective patterns. This rationale aligns with the authors of [20,21], who emphasize that saturation is achieved when no new relevant dimensions emerge from the data, rather than when a predetermined number of interviews is reached.

In terms of selecting participants, the last researcher of this study selected the first participant for the study through a social connection facilitated by his life partner, who worked with this Spanish nonbinary nurse that had recently become a parent. The three of them initiated a conversation about the study’s purpose via WhatsApp, building a sense of involvement and shared interest.

Once the first researcher conducted the first interview, transcribed, and analyzed it, we wanted to contrast the findings by interviewing a second participant. The first participant told us that she had found references for her pregnancy in the Instagram hashtag #nonbinarypregnancy, which made us determined to find a new participant through this channel. After sending a message to two US-based profiles, the first researcher found a new participant quickly. The first one who responded met the inclusion criteria and made themselves available for interview.

### 2.3. Data Collection

The first researcher interviewed each participant, collecting data from October 2022 until April 2023, through two semi-structured interviews for each one, administered through online video calls due to physical distance and scheduling differences. The interviewer audio-recorded, transcribed (with assistance from Word 16 software), and analyzed the first interview before arranging the second one.

The interviewer previously provided both participants with an information sheet to give us their informed consent to participate in the study. Each of the first round of interviews for both participants had a duration of 40 min, whereas the first participant’s (P1) second interview lasted 30 min, and that of the second participant (P2) lasted 40 min (the first researcher (interviewer) decided to conduct a second interview with each participant to further explore the phenomenon after her intuitive and preliminary analysis of the first encounter).

In each interview, the interviewer asked specific questions relevant to the topic regardless of conversational fluidity with diverse open-ended questions. The interview guide was designed by the authors based on a literature review on the emotional experiences of nonbinary individuals and adapted to a hermeneutic approach. It included open-ended questions aimed at exploring emotions, identity, and parenting experiences. In the first interview, she asked open-ended questions (preceded by “How does living a nonbinary parenthood make you feel?”), while in the second, she asked more detailed or specific questions. These questions are converted into Table 1.

### 2.4. Analysis

Three of the researchers coded the data (the first, second, and last one, respectively), performed a hermeneutic analysis using Nvivo 14 qualitative analysis software, and followed Ricoeur’s criteria [16,22] as follows:

Once the first researcher/interviewer finished the P1 transcriptions, she and the two other coders did an in-depth reading to fully comprehend the text and select its main ideas, enabling the structure of an intuitive explanatory framework. Then, they initiated the three levels proposed by Ricoeur [16,22].

At the first level (explanation), the researchers selected nodes—or units of complete meaning (meaning, explanation, and justification)—related to the starting categories (the main galaxies of the Universe of Emotions taxonomy) [13]. In doing so, they revised the text to verify that did not overlook any relevant meaning. After that, they created 67 analytical subcategories linked to the starting ones and triangulated these results with the fourth researcher.

Although less internationally known than models such as Plutchik’s Wheel of Emotions or Ekman’s six basic emotions [23,24], Bisquerra’s taxonomy [13] was selected for its pedagogical clarity, its alignment with the hermeneutic framework of this study, and its emphasis on emotional granularity, particularly useful for analyzing participants’ affective discourse. Notably, Bisquerra’s model builds upon foundational contributions from earlier taxonomies while expanding their scope to include a broader range of emotional categories, such as social and ambiguous emotions, and a metaphorical structure that enhances its interpretive potential. The taxonomy organizes emotions into five broad categories—positive, negative, ambiguous, primary, and social—allowing for a nuanced and culturally resonant analysis. Moreover, this model has been successfully applied in several peer-reviewed international publications in English, particularly in hermeneutic and affective research (see the Supplementary Materials). These precedents support the relevance and applicability of Bisquerra’s taxonomy beyond Spanish-speaking contexts. An explanatory summary of the taxonomy is provided in the Supplementary Materials.

At the second level (naïve understanding), the researchers reorganized the nodes according to the subcategories previously established. Once grouped, the nodes were further refined based on semantic affinity, allowing for a higher level of abstraction and the identification of recurring patterns. This process led to the construction of more abstract intermediate categories. For instance, codes such as “institutional rejection”, “legal invisibility”, and “frustration with the healthcare system” were consolidated under the broader category of “administrative dislocation”. At this stage, the researchers also began coding the transcripts from the second participant (P2), linking each new node to existing codes or creating new ones when novel meanings emerged.

At the third level (in-depth understanding), the research team deepened their interpretation of the participants’ emotions through Ricoeur’s hermeneutic arc. This phase involved a critical evaluation of their pre-understanding of the phenomenon and a thorough rereading of the data corpus, leading to the highest level of abstraction. The previously constructed categories were integrated into broader interpretive themes—such as “Clearly, you don’t fit, so…”—which articulate emotional and existential trajectories. This process was iterative and reflexive, involving triangulation among three coders and validation with both participants and external reviewers.

### 2.5. Rigor

Since the development of the study, the research team followed Calderon’s quality and rigor criteria for qualitative studies [25] (Table 2).

### 2.6. Ethical Considerations

Concerning the confidentiality and anonymity of the participants, we assigned P1 as an acronym for Participant 1 and P2 for Participant 2. We conducted the study in compliance with the principles of the Declaration of Helsinki, and it was approved by the Ethics Committee for the Province of Cordoba (Spain). The first researcher received verbal and written consent after providing detailed information about the study to the participants. The research team processed the personal data following the General Data Protection Regulation EU/2016/679, of 27 April 2016, and the provisions of the Organic Law 3/2018, of 5 December, on the Personal Data Protection and Digital Rights Guarantee of Spain.

All interviews were audio-recorded with participants’ informed consent and subsequently transcribed verbatim. Personal identifiers, including names, locations, and any potentially identifying details, were removed and replaced with pseudonyms during transcription. The anonymized transcripts and audio files were stored on encrypted, password-protected devices accessible only to the research team. These measures were taken to ensure the confidentiality and safety of participants, particularly given the small size and potentially identifiable nature of the sample.

## 3. Results

P1 (she/they/them) was a 32-year-old white Spanish nurse, assigned female at birth, with more than eight years of cohabitation with their cisgender heterosexual male partner (in a relationship that *most people consider a heteronormative one*, quoting their words) and an *adre*—a word in Spanish that has no gender marking, such as *padre* (father) or *madre* (mother)—of two one-year-old toddlers. They identified as a *nonbinary person* (P1 understands this identity *within the trans label*, as they put it) during the past seven years. P1 felt (quoting their words) *minimal gender dysphoria*. At the moment of the interview, they lived in an urban area in the south of Spain.

P2 (he/they/them) was a 34-year-old white North American physiotherapist, born in Washington State, assigned female at birth, a current *doula* and former soldier, married for five years with their husband (cisgender homosexual male partner, sharing *a visibly queer relationship*, quoting their words), and a parent to a ten-year-old child (their current partner is not his biological dad). Their child called them *mum* (and, at times, *legal guardian*), while his friends called them his *dad*. P2 identified themselves as (quoting) a *transmasculine nonbinary person*. They have undergone GAC—or Hormonal Replacement Therapy (HRT), as they put it—and the expression “gender dysphoria” was more frequent in their interviews, expressing more gender dysphoria than P1. At the moment of the interview, they lived in a suburban area in the state of Texas.

**Figure 1:**
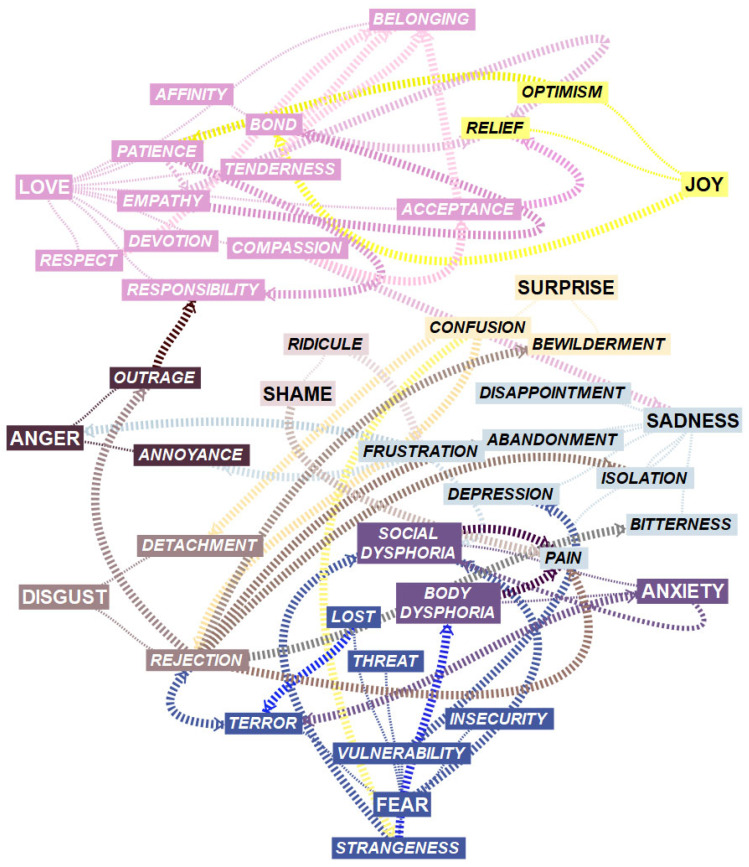
Emotional universe of nonbinary parents.

Through our analysis, we identified associated emotions that could be grouped accordingly into four themes, including: (1) *A story of misunderstanding: “What are you [and what am I], a combat helicopter?”*; (2) *Clearly, you don’t fit, so…*; (3) *But (a new) family is there*; (4) *No monster here: I’m, at the core, a human being* (Table 3). These themes describe different trajectories within Bisquerra’s emotional galaxies [13] (Figure 1).

(1) **A story of misunderstanding: “What are you [and what am I], a combat helicopter?”**

P1 and P2 shared a common personal history of embodied and social dysphoria. Both received a cisnormative education from their parents, which created a complex relationship with their bodies and their close social environment since childhood. They felt something in them was *not right* and did not understand what was wrong. Feeling this otherness, it is not surprising that they felt insecure, both emotionally and socially, in their childhood and adolescence:

*“Well, I just used to say: ‘I’m a little broken’ […] It caused me so many personality problems”.* (P1)

As they grew older, the problem, far from being fixed, intensified, and they suffered emotions linked to anxiety and depression, reaching the point of feeling lost in their lives:

*“I had so much depression when I was younger, a lot related to the gender stuff”.* (P2)

Thus, when they became pregnant, they could not help but feel a jolt of terror at seeing themselves as so feminine because of the physiological changes in the process:

*“Pregnancy is a whole other thing, because your entire body changes, your breasts get bigger, not being able to see your toes is even complicated for cisgender women…”.* (P1)

For all these reasons, the participants were well aware that understanding themselves and their nonbinary gender identity was not easy—especially with their pregnancy and its accentuated perceived feminine traits. Cisheteronormative society viewed their pregnancy as confirmation of their *womanhood*, and now more than ever, any other consideration of that identity was simply ridiculous and out of place:

*“They make it sound ridiculous: ‘Wow how many sexes and genders are you?’, ‘What are you, a combat helicopter?’ […] Pregnancy is a whole different story because your body changes so much, and if it’s already complicated for some cis women… imagine for me. [...] I didn’t enjoy pregnancy in any way”.* (P1)

They always lived under the threat of mockery, of getting rude transphobic reactions aimed to degrade the acceptability of trans and nonbinary identities and, they confessed, they struggled to go unnoticed (but how to do so with a pregnant belly!) or to integrate so as not to feel rejected:

*“I tried really hard to conform because I did get made fun [of] and gender was a big part [of it] […] I tried really hard to be a girl… I grew my hair out long, I tried to wear makeup… I tried really hard to pretend to be a girl… maybe I was wrong, maybe I was supposed to be female… it was rough”.* (P2)

However, rejection was present in their lives and was associated with disappointment and frustration. In this sense, P1 said feeling these emotions related to the social and health care provided to nonbinary people since, according to them, they needed a diagnosis to receive GAC:

*“There are nonbinary people that want hormonal therapy, but they can’t as they lack a transgender diagnosis”.* (P1)

Meanwhile, P2 felt these emotions due to what they believed was the lack of visibility, support, and acceptance of his identity, even within the LGTBIQ+ community:

*“The acceptance levels go: lesbian>gay>trans (binary)>nonbinary”.* (P2)

(2) **Clearly, you don’t fit, so…**

Beyond the socio-health field or LGTBIQ+ community, the participants also do not feel they fit in with the local and national administration, where (both say) they do not have full administrative recognition. P2 remarked that in the state where he lived at the time of the interview, Texas, the administration did not recognize their identity in legal documents:

*“The State of Texas does not recognize nonbinary identities so I must pick one; I guess it will be male”.* (P2)

For their part, P1 went beyond the absence of nonbinary options by pointing out, with bewilderment, the absurdity of the obligation to define oneself legally in a binary way. What is the point of doing it this way if we all are people first and foremost?

*“I believe indicating ‘man’ or ‘woman’ on your ID is pointless. What is the point in saying that when applying for a job if we are supposed to be equal?”.* (P1)

However, the dominant cisheteronormative culture was stubborn in categorizing gender in a binary way and, obviously, in categorizing all people in this way, leaving no room for any other possibility, to the annoyance and frustration of our participants. Both felt victims of social disdain for their gender self-determination:

*“They try to put you in one of the two boxes: square or circle, and they really don’t get you are a triangle […] People don’t even know what a trans person is. […] If they don’t know what a trans person is, how are they going to know what a nonbinary person is?”.* (P1)

*“People are definitely quite dismissive when they don’t understand how committed gender is to your identity”.* (P2)

In addition, when people showed interest, they again felt annoyed and frustrated at the failed attempts to understand their identity:

*“Sometimes I tell them to go on Wikipedia and read because I can’t be bothered to explain as there’s too much”.* (P1)

At certain times—at work, in their neighborhood, and even at home—they transformed the annoyance and frustration caused by the abovementioned disdain and incomprehension into outrage in the face of rejection. P1 mentioned how, from time to time, in the hospital where they worked, they heard very transphobic comments by colleagues who, moreover, assumed that all the people present (when they talked about them) were cisheteronormative:

*“[At work] you hear transphobic comments from colleagues [...] and you say, ‘What is this bullshit this person is saying?!’”.* (P1)

P2, on the other hand, felt abandonment, isolation, vulnerability, pain, and bitterness in the face of the rejection, represented by the fact that their neighbor would not let her children play together when she found out about their identity:

*“I told my neighbor while our kids were playing, I was transnonbinary and she said: ‘Oh well it’s OK, I don’t hate the sinner I just hate de sin’. She never brought her son back to play again”.* (P2)

This participant added disappointment and bitterness to the aforementioned emotions when they saw how their ex-partner wanted to impose that they reject GAC and, at the same time, the end of any conversation about it:

*“After mentioning my ex-husband the desire to start HRT he said: ‘no, you are not and we will not talk about this again’”.* (P2)

(3) **But (a new) family is there**

In the face of this ignorance and social rejection, the participants felt the need to find a way to become different reference figures for their children. P1 and P2 needed to exert another way of parenthood. In this other way, they knew how to put themselves in the place of their mothers, through empathy, to recognize (as P1 stated) how she raised them in the way that society considered to be the best, understanding that—in terms of gender—it was not only the best but also the only way:

*“My mum had never read about this, so she tried to educate me like her parents did, in the way she thought was correct”.* (P1)

From an alternative parenting perspective, P2 acknowledged their mother’s efforts to respect their identity, respect represented by the use of the pronouns chosen by the person who, at another time, was her daughter:

*“My relationship with mum is a giant mess due to other issues, but she knows and tries with pronouns and usually gets them right”.* (P2)

When P2 found the previously mentioned empathy and respect in their son’s behavior towards them, they could do nothing but experience a strong feeling of love and tenderness. Their son was beginning to know P2’s gender identity, respect it, and, at the same time, not give it more importance than other things they could provide, such as (for example) playtime:

*“When I originally came out like we read a book about pronouns, and we read a book about gender identity and then like I came out to him after that, and he just wanted to play Minecraft he did not care at all”.* (P2)

The participant realized that their son respected them to the point of addressing him with what, a priori, was a legal, cold term—like *legal guardian*—but which he turned into a sign of love and consideration:

*“At one point he called me ‘legal guardian’, and I told my husband he was doing that because it’s genderless; he is not trying to withdraw from us, he is trying to be respectful of who I am”.* (P2)

*[As P1 noted] “They’re going to love me anyway because I’m their maternal… or paternal figure, whatever you want to call it”.* (P1)

In their parenthood, their children were their main priority. Although both were aware of the position of vulnerability and disempowerment in which they were placed socially because of their gender identity, they were sensitive to the reality that their children were even more vulnerable. For this reason, they prioritized their needs over their preferences. For example, P2 respected the way their son addressed them, and P1 (with their children still very young) delayed breast reduction surgery (*top surgery*) because of the inconvenience it might cause for parenting:

*“I’m just letting him the lead that not yeah like it would be cool if eventually he started calling me something else [other than mum or dad] but I mean it’s-it’s his call like because… I think the power dynamic between parent and child is such that I have the responsibility to put him first”.* (P2)

*“Until they’re 3 years old I won’t undergo top surgery, being uncapable to hold them… right now isn’t the moment”.* (P1)

Among the values of this new way of parenting is that their children should have *agency*, particularly regarding gender identity. P1 is optimistic that society will, eventually, assume this value and that neither the knowledge of different identities nor that of gender self-determination will be a problem for their children:

*“My kids will simply have an education where they’ll know the different gender identities, so they place themselves wherever it is meant to be”.* (P1)

On the contrary, when P2 referred to the value of autonomy, they did so from compassion and pain when they gave an example of how their son’s decision around being able to speak openly about who *his legal guardian* is could cause him to be rejected and isolated by his peers:

*“I told him he needs to understand that if kids at school know I’m transnonbinary their parents might not let them play with him, and that was his call”.* (P2)

In the same way that they sought to exert a new way of parenthood, they also sought to integrate themselves into a new chosen family where the feeling and behavior of acceptance and respect would be there daily. But, when this did not happen, it was not traumatic to break ties, to detach from certain people who, either by blood or legal ties, were part, in the past, of their official family. Thus, P1 expressed an affinity for specific members of their family of the same generation. In contrast, they did not feel this affinity towards those of previous generations—including their parents—to the point of preferring to hide this vital part of themselves in order, they said, to not hurt them:

*“My sister and some cousins know as opposed to my parents and uncles, they wouldn’t understand, and I don’t wish to feel bad”.* (P1)

P2, however, showed without qualms who they were: if their family accept it, that is superb, but if they do not, it is OK. In their case, he felt this detachment from anyone who did not accept him, thanks mainly to moving from his home state of Washington to Texas years ago:

*“My family all know. The ones who aren’t accepting are no longer a part of my life. I moved far away, so it’s not really an issue”.* (P2)

(4) **No monster here: I’m, at the core, a human being**

The love and acceptance they manifested from their parenthood went beyond pronouns. Thanks to their feelings, the participants connected with an identity transcending the nonbinary one: *human people*. Thus, when P2 heard their son call them mum, they did not give it any importance (the same as P1 with feminine pronouns):

*“[Sometimes] he [their son] refers to me as mum and his friends ask: ‘do you mean your dad?’ […] There’s just like so much more flexibility… you can be a feminine guy and you can be a masculine woman and then you could just be nonbinary and then just be whoever you are. And not have to worry about so much”.* (P2)

*“In the end, I have spent my whole life with the feminine pronoun, it’s mine, and it doesn’t cause me dysphoria […] I am who I am, and I accept myself; I accept myself”.* (P1)

The emotional journey of P1 and P2 after exercising parenthood in the aforementioned way and creating a new family led to emotions linked to love, respect, and acceptance. However, (attenuated) body dysphoria remained a reality in both of their lives. Both claimed to need breast reduction surgery. The use of non-surgical aesthetics (either through make-up or body shaping through exercise) and GAC were not considered sufficient:

*“I can get to what I want through makeup, going to the gym, exercising, and, at some point, as planned, through surgery”.* (P1)

*“Being on HRT has definitely helped a lot, but I do need top surgery to be comfortable”.* (P2)

In this attenuation of their body dysphoria, their current life partners have played a significant role. P1 felt supported in their identity—always and naturally—by their cisgender heterosexual male partner:

*“We have been together for over eight years; I didn’t tell him straight away, but he knows, and he is okay with it […] I wouldn’t be with someone intolerant”.* (P1)

P2, on the other hand, said that with their partner, a cisgender homosexual man—they did have to work together on acceptance, which did not prevent him from loving them, bonding closely with their son (from a previous partner), or defending their love to skeptical friends:

*“At first, it was a bigger adjustment for him than for me, but he is super involved with my son, and he also protected me, even from his friends […] He has tried from the very beginning to be supportive… now he is absolutely my biggest supporter”.* (P2)

Most of their peers in their current and respective jobs, as well as some spaces within social networks, such as Instagram, where they found other nonbinary references, also contributed to acceptance and alleviated the feeling of alienation. Thanks to all of these influences, the participants felt they belonged:

*“After all I feel good because as a person, as P1, they treat me well”.* (P1)

*That job was very queer, they were all very inclusive and supportive except for one person* (P2).

*“Look, if you put in Instagram ‘nonbinary pregnancy’ [in English], like this, with the hashtag, you will get a lot of people”.* (P1)

## 4. Discussion

The first interpretation that we make of the emotional universe of nonbinary parents has to do with that of a journey: through its different galaxies and stars, we move from emotions linked to the Galaxies of Fear, Anxiety, and Sadness to others related to the Galaxies of Joy and Love. From one place to another in this universe, the participants get to know other emotions related to disgust, anger, or surprise. Still, it is noteworthy that at no time do they encounter emotions included in the Galaxy of Happiness. With the present discussion, we want to finish understanding the different circumstances that make this journey possible, both with their present and absent emotions.

In this regard, while the analysis identified a set of clearly articulated emotions expressed by the participants, it is also important to consider those emotions that were absent or emerged in ambiguous or conflicting ways. For example, none of the interviewees explicitly mentioned emotions such as euphoria, fulfillment, or happiness, all belonging to the Galaxy of Happiness in Bisquerra’s taxonomy [13], which may suggest that unresolved emotional tensions persist even in contexts of acceptance. Likewise, some emotions, such as relief or acceptance, were expressed ambivalently, coexisting with feelings of bodily dysphoria or institutional frustration. This ambivalence suggests that the emotional experience of nonbinary parenthood is neither linear nor homogeneous but instead marked by affective contradictions that deserve deeper exploration. In this sense, the silence or absence of certain emotions also constitutes meaningful data that enriches the hermeneutic interpretation of the phenomenon.

Within each galaxy’s affective complexity, the researchers wish to highlight three key emotions: strangeness, belonging, and acceptance. We found the first at the beginning of the journey, the second at the end, and the third is the affective experience that allows transition in both a broad and a specific sense.

In the beginning, P1 and P2 felt alienated, with no possibility of connection, either with themselves or with others, with a body in which they did not see themselves identified in, and with a society in which they did not feel included. Hence, we obtain the set of emotions we associated in the results with the theme *A story of misunderstanding*. However, the affective landscape is entirely different at the end of their journey. In *No monster here*, the participants felt they belonged because they had a family, a work group, and a virtual community in which they felt accepted.

Therefore, although in the discourse of P1 and P2, we cannot point to any of the emotions in the Galaxy of Happiness—such as wellbeing, tranquility, or balance—we do highlight belonging (inside the Galaxy of Love) as the closest one.

Going back to the beginning, we associate the feeling of strangeness (characteristic and fundamental in the first theme) with what Todres et al. [26] call *dislocation*, a dimension of dehumanization in which “people are challenged to find a sense of place in a new and unknown culture where norms and routines are alien to them” (p. 73). We interpret this link between strangeness and displacement with a double value. On the one hand, it helps us to understand the experience of the participants when they feel that they do not fit into either the locus of their body (body dysphoria) or the locus of the community (social dysphoria)—that is, they do not find their place in either their body or the social structure. On the other hand, the journey from strangeness to belonging is also a journey from dehumanization to humanization. The emotional transition from a sense of strangeness to a sense of belonging can also be understood through Minority Stress Theory, where social and familial acceptance protects against the chronic stress caused by discrimination [11]. Likewise, from the perspective of Queer Theory, this transition can be interpreted as a subversion of dominant gender norms, where emotional experience becomes an act of resistance and identity affirmation [12].

However, until they reach that point, they are left with complicated stages of the journey, including anxiety, depression, and terror. While we find many examples of trans people’s experiences of anxiety and depression in the scientific literature [14], the same is not true when we look for experiences of body dysphoria or the terror of being pregnant in nonbinary people. It was P1 who offered us, in the first of their interviews, the opportunity to get closer to this phenomenon by reading the graphic novel entitled “*Pregnant butch: nine long months spent in drag*” [27]. In this autobiographical fiction, the author recounts how becoming pregnant meant coming into conflict with her own identity, with gender stereotypes, and with a cisheteronormative society [28].

Participants reported that the body and social dysphoria they experienced were most significant during the physiological development of pregnancy. They felt ridiculous and, at the same time, threatened by people’s jokes and disregard. With their new body, it was difficult for them to pass unnoticed or to stay integrated, and we have found no examples in the evidence on passing or concealment of nonbinary people [29] which allude to this circumstance when they are pregnant.

The participants were confronted throughout their lives and even more so during their pregnancy with the paradox of visibility [12]. Becoming visible was a way of vindicating themselves and, at the same time, a trap that exposed them to different forms of violence. Rejection, disappointment, and frustration are very present in two particular moments: when P1 understands that, in Spain, with an assessment that ignores the nonbinary identity, one cannot access the GAC, and when P2 assumes the marginal character that the nonbinary identity had, even within the LGTBIQ+ collective.

The Spanish healthcare system offers care to the trans population “in both centralized (by one interdisciplinary institution) and decentralized settings (by different medical institutions spread over several locations)” [30] (p. 7), and from these services, GAC can be accessed once their specialized medical teams assess gender dysphoria, following the guidelines of the so-called Trans Law [31]. However, this law does not mention at any point the nonbinary identity and, recently, the highest representative in matters of equality and LGTBIQ+ rights of the Spanish state denied that, in the current legislature, the nonbinary gender would be officially recognized in the country [32]. Therefore, for any service, including access to the GAC, nonbinary people in Spain may be excluded because they are nonbinary, as the results of Gómez-Ibáñez et al. [33] confirm.

Regarding the marginal nature of the nonbinary gender within the LGTBIQ+ community in the healthcare field, Newsom et al. [34] stated that due to cisnormative biases, “within the LGBTQ+ community, the transgender and nonbinary […] population experience a disproportionate amount of discrimination when seeking health care” (p. 453). Even in the virtual context, Devito et al. [35] seem to support P2’s words when they state that “on platforms that do exist for sapphics, transgender women and nonbinary people are often subject to discrimination, fetishization, and stigmatization” (p. 203:1).

The participants also felt rejected when they had to identify themselves to the administration system of their countries (in the case of P2, to the administration of the state in which they were currently living), even though so-called *misgendering*—or the use of the wrong pronoun or gendered language to mention someone—is recognized as bad practice because of the adverse impacts on the mental health and wellbeing of trans persons [29]. Clary et al. [36], in their study of trans and gender-diverse people conducted in Texas, found that they felt invalidated when they engaged in some settings, mainly when languages were very gendered, among other elements of Texas culture, especially patriotic, masculine, or Christian symbology.

In Spain, and from their nonbinary logic, P1 felt bewilderment at the administrative obligation to identify themself binarily. In our opinion, this emotion is easy to understand when countries or states absurdly exercise the so-called symbolic violence—that which is deceptive and happens consistently throughout everyday life [37]—through the misgendering of their non-inclusive administrative language. This fact feeds back into their feeling of strangeness and dislocation.

However, other less subtle forms of violence were also present in the lives of P1 and P2 in the form of public disqualifications of trans identity and coercion by former romantic partners, generating the emotions mentioned above of outrage, pain, and bitterness, among others. In Andalusia, the Spanish region with a solid Catholic tradition where P1 lives, there has been an increase in hate speech in recent years explicitly directed at trans people, according to the Observatorio Andaluz contra la Homofobia, Bifobia, y Transfobia [Andalusian Watch against Homophobia, Biphobia, and Transphobia] [38]. In addition, Kurdyla [39] shows evidence of how trans and gender-diverse people endure high rates of intimate partner violence globally.

From the theme *But (a new) family is here* onwards, P1 and P2 move towards a much brighter space in their Universe of Emotions, namely towards the Galaxy of Love. Parenthood is here the main milestone. Before this, the participants emphasized their nonbinary identity in their set of identifying features, whereas now, they can dilute it (without renouncing it) thanks to their new status as parents. By turning their nonbinary identity into just one part of their blended total identity Meyer [11], they can increase their emotional wellbeing.

Both now prioritize caring for their children, educating them about affective, sexual, and gender diversity, and the value of autonomy, in general, and when it comes to choosing and respecting different identities. Similarly, P1 and P2 exercise this autonomy in the emotional field when they know how to distance themselves from family members who do not accept them. Both seem to be aware of “the importance of parenting quality, positive coparenting, and parent mental health for child outcomes” as Goldberg [6] (p. 4) notes in his review of LGBTQ-parent families.

In our study, the concept of acceptance operates at both personal and social levels, and these dimensions are deeply intertwined. While acceptance is a universal psychological need, for nonbinary individuals—as Meyer’s Minority Stress Model suggests [11]—personal acceptance is particularly dependent on social recognition due to the chronic stressors associated with stigma and marginalization. Parenthood seems to facilitate the social recognition and feelings of self-acceptance that the participants enjoyed during the interviews. According to Malatino [12], social recognition is grounded in gender, but we wonder if the recognition they enjoy is partial, as it seems mainly motivated by their parenthood. In our study, parenthood emerges as an emotional turning point that facilitates experiences of acceptance and belonging. However, it is worth asking whether this recognition is truly unconditional or, to some extent, shaped by normative expectations about what it means to be a “good father” or “good mother”. In some cases, social validation may depend on the ability to fulfill socially valued roles, such as caregiving, suggesting a form of instrumental rather than full acceptance. This idea resonates with Malatino’s reflections on the conditional nature of recognition for trans and nonbinary individuals [12] and Butler’s argument [40] that recognition is differentially allocated based on compliance with dominant gender norms. In this sense, parenthood can act as a catalyst for acceptance and a mechanism of symbolic regulation. The absence of emotions such as euphoria or fulfillment, even in contexts of familiar acceptance, could be interpreted as an indication that this acceptance remains fragile or conditional. Future research could explore how this acceptance is negotiated in contexts where parenthood does not conform to traditional models, or when it intersects with other dimensions such as racialization or social class.

We believe that if there is partial recognition, it may come (according to the study results) from their work environment, but not from their life partners or their children, in which case recognition is total. This being seen in its entirety was also experienced in virtual communities, specifically with those related to nonbinary pregnancy. However, they did not mention other hashtags, such as nonbinary parents. In the same vein, we found different studies that referred to nonbinary parents’ pathways to parenthood [6,15,41], but very few that addressed nonbinary parenthood once the children were born.

The fact that they feel seen and accepted favors their self-acceptance and sense of belonging. However, these findings are situated within a broader clinical context marked by distrust and lack of knowledge regarding clinical guidelines for supporting trans and nonbinary individuals, particularly in primary care [3]. This distrust and lack of understanding can lead to contradictory emotional experiences, where unequal or misinformed clinical practices strain the pursuit of recognition. In this regard, the critique by Pérez-Álvarez and Errasti [4] of potential premature medicalization and the ideological framing of gender discourse broadens the hermeneutic analysis of emotions, acknowledging that acceptance or rejection stems not only from the immediate social environment but also from clinical structures that mediate access to care and identity validation.

Returning to the humanization dimensions of Todres et al. [26], P1 and P2 go from dislocation to feeling—thanks to their parenthood—that they inhabit a particular place where they find “the kind of belonging that provides a degree of security, comfort, familiarity, continuity, and unreflective ease” (p. 73), a place that belongs to them because, after all, they are, at the core, a human being.

## 5. Strength and Limitations of the Work

The study’s limitations are mainly due to the cisgender identity of its researchers. From our etic perspective, we can facilitate understanding of the emotional complexity of nonbinary parents by the cisgender community. However, the transferability of the results to nonbinary parents would be greater if we had an emic perspective. Furthermore, although all the researchers share the cultural context of P1 by living in the same city—and despite being aware of the cultural parallels marked by the religious tradition of southern Spain and the southern U.S.A. and by the context of cultural globalization facilitated by social networks and the global hegemony of the English language (P1 followed English accounts on social networks and consumed American cultural products, such as the one mentioned by Summers [28])—none of us have made a prolonged stay in the United States, which distances us, even further, from the emic vision mentioned. Likewise, only one researcher out of the four is familiar with parenthood. In an attempt to alleviate these limitations, the researchers relied on the collaboration of two nonbinary persons external to the study (a scholar working at a Spanish university and another with a U.S. Bachelor of Science degree in Linguistics) who reviewed the final manuscript, suggesting corrections and adding comments. In addition, all of the researchers are allies of the LGTBIQ+ community, in general, and of the trans community, in particular; the lead researcher is bisexual, while the last researcher has a female gender expression with a nonnormative body for which he/they feels body dysphoria and has, at different times, resorted to concealment strategies to accept himself and feel accepted, circumstances that facilitate the empathic imagination to approach the phenomenon under study.

In addition to the positional limitations of the research team, we also acknowledge the potential for selection bias in our sampling process. The participants’ willingness to share their experiences, their digital literacy, and their engagement with online platforms may have shaped the narratives collected, privileging voices that are more open, articulate, and digitally connected. These factors may limit the inclusion of nonbinary parents who are less visible or less comfortable in digital or research settings.

In this sense, while the insights generated are deep and contextually grounded, they are necessarily narrow. The study does not aim to offer generalizable conclusions about nonbinary parenthood as a whole, but rather to illuminate specific affective and discursive patterns within a particular sociocultural and experiential frame. Future research should seek to broaden this scope by incorporating more diverse and intersectional voices.

Furthermore, the emotional taxonomy we have used to explore the participants’ emotions is recent, little known and, as few studies published to date have used it, it may be difficult to understand for those unfamiliar with it.

Finally, the size of the sample, which we determined by the saturation criterion based on what we considered essential and novel about our research interest, is insufficient to collect all the dimensions of the affective discourse of nonbinary parents, needing, for instance, a more intersectional and inclusive perspective in future studies. It is important to acknowledge that the findings presented in this study are context-specific and reflect the lived experiences of two white, middle-class, nonbinary parents from Spain and the United States. As such, they do not capture the full diversity of nonbinary parenting experiences, particularly those shaped by intersecting systems of marginalization such as racialization, disability, migration status, or socioeconomic inequality. While the depth and richness of the narratives offer valuable insights, future research should aim to include a broader and more intersectional range of voices to better reflect the heterogeneity and complexity of nonbinary parenthood.

Nevertheless, our intention as researchers was to approach the phenomenon relying on the richness of the obtained data, without the aspiration of declaring all, the only, or the last points about the phenomenon.

In light of the limitations discussed, we recommend that future studies on nonbinary parenthood actively involve nonbinary researchers in the design, data collection, and analysis phases. Their participation would not only contribute to a more emic and contextually grounded interpretation of the data but also help to mitigate potential biases stemming from cisnormative assumptions. This collaborative approach would strengthen the ethical and epistemological foundations of future research in this field.

Moreover, future studies could benefit from the inclusion of standardized instruments to assess emotional wellbeing, identity development, or social support in nonbinary parents. The integration of such measures—either within mixed-methods designs or in larger-scale quantitative studies—would allow for broader comparisons across populations and contexts, and contribute to the generalizability of the findings. While our study prioritized depth and contextual richness, we recognize the value of complementing qualitative insights with more systematic and scalable approaches in future research.

## 6. Conclusions

In the emotional universe of nonbinary parents, there were three key emotions: strangeness, belonging, and acceptance—the first at the beginning of their journey, the second at the end, and the third the emotional experience that allowed their transition in both a broad and a specific sense. In the beginning, these parents felt alienated, with no possibility of connection, with themselves or with others, with a body in which they did not see themselves identified with, and with a society in which they did not feel included. Hence, we obtain the set of emotions we associated in the results with the themes *A story of misunderstanding* and *Clearly, you don’t fit, so*—such as body dysphoria, confusion, insecurity, anxiety, depression, terror, outrage, rejection, abandonment, isolation, and bitterness. However, the affective landscape was entirely different at the end of their journey; through the last two themes (*But (a new) family is there* and *No monster here: I’m, at the core, a human being*), there were feelings of empathy, respect, love, tenderness, responsibility, devotion, respect, bond, patience, optimism, relief, and compassion. Eventually, the participants felt they belonged because they had a family, a work group, and a virtual community in which they felt accepted. Their journey from strangeness to belonging is also a journey from dehumanization to humanization—with their experience of parenthood as a catalyst for this transition.

## Figures and Tables

**Table 1:** Main questions belonging to the semi-structured interview.

*Primary questions related to the main topic*
When did you realize you were nonbinary, and how did you experience this? Do you feel comfortable with the term “mother”/“motherhood”? Why? Have you ever felt mistreated by work colleagues/family/friends? How so? How do you self-identify, and what pronouns do you prefer? Why? Can you tell me a bit about yourself and your childhood?
*Derived and spontaneous questions*
How is your relationship with your partner? … And with your family, do you feel accepted? Do you see any differences between the advantages of being trans compared to being nonbinary regarding society? Do you consider yourself a generally confident person, especially regarding your identity? How so? Have you had any confronting encounters with people surrounding you that have caused any emotional impact? How is your relationship with your body?

**Table 2:** Study quality and rigor criteria.

*Theoretical, epistemological, and methodological appropriateness*	The hermeneutical approach, which interprets and comprehends the exposed topic, allows us to explore the participants’ emotions and the realities they face throughout their lives and nonbinary parenthood. It is highly relevant to create a base of comfort and trust for a better narrative outcome, as investigators also commit to an emotional journey and need to leave aside prejudices and disagreements.
*Relevance*	Scientific research has focused mainly on anxiety and depression suffered by nonbinary people and their different pathways to becoming parents. However, very little work has focused specifically on the integral emotional experiences of nonbinary parents. An effort is required to take a thorough approach to the emotional experiences of nonbinary parents, bearing in mind that through negative and positive emotions, we can obtain robust insights into these parents’ personal and social contexts. Therefore, we can facilitate the understanding of the emotional complexity of nonbinary parents to the trans and cisgender communities. Through this understanding, nurses and the organizations they work for can improve their competence in their holistic care. Acceptance from nonbinary parents’ social contexts, of which nursing is a part, is a critical factor in their health and emotional wellbeing.
*Validity*	The researchers considered the study’s validity through a double triangulation between the lead researcher and participants and between the research team. In this sense, they returned the resulting (verbatim) transcripts to the participants for validation and comment. The last and second co-researchers assisted the first during the investigation, independently analyzing the study’s corpus. In addition, after finishing each level of analysis, the three agreed on their judgments and shared them with the third researcher. Eventually, after the third level of analysis, the team shared their findings with the participants. Nonetheless, the researchers completed the hermeneutical arch of the third level of analysis with the development of the final explanatory framework of results and with the discussion of the study results. In addition, two nonbinary people external to the study (a scholar working at a Spanish university and another with a U.S. Bachelor of Science in Linguistics degree) reviewed the final manuscript, suggesting corrections and adding comments. All in all, the research team provided a detailed description of the study setting to ensure the findings’ transferability (or the degree of applicability to different contexts, circumstances, and participants).
*Reflexivity*	All researchers are nurses and are notably motivated and keen on the psychosocial factors of nursing care and research, particularly the associations between diversity and emotional health. On the other hand, all four researchers are cisgender and allies of the LGTBIQ+ community, in general, and of the trans community, in particular. In addition, the lead researcher is bisexual, while the last researcher has a female gender expression with a nonnormative body for which he/they feels body dysphoria and has, at different times, resorted to concealment strategies to accept himself and feel accepted. Additionally, the whole research team is fluent in Spanish and English since the first researcher has double nationality (Spanish and British), and the rest (being native Spanish speakers) have an accredited level of English (C1).

**Table 3:** Analysis themes, subthemes, and related emotions.

*A story of misunderstanding: “What are you [and what am I], a combat helicopter?”*	Seeing myself so feminine was terrifying	Body dysphoria Confusion Strangeness Insecurity Anxiety Depression Lost Terror
	Understanding (and caring for) the nonbinary identity is not easy	Social dysphoria Ridicule Threat Rejection Disappointment Frustration
*Clearly, you don’t fit, so…*	We don’t have fully administrative recognition	Rejection Frustration Bewilderment
	People ignore a lot about us...	Annoyance Frustration
	… And (what a surprise!) they reject us	Outrage Rejection Abandonment Isolation Vulnerability Pain Disappointment Bitterness
*But (a new) family is there*	There is another way of parenthood…	Empathy Respect Love Tenderness
	… where children are the priority	Responsibility Devotion Respect Bond Patience
	My children’s relationship with gender identity is gonna be… their call	Optimism Relief Respect Compassion Pain
	If family accept it, that’s superb. If they don’t, it’s OK.	Acceptance Respect Detachment Affinity Empathy
*No monster here: I’m,* *at the core,* *a human being*	Parenthood love beyond pronouns	Acceptance
	I (almost) embrace my body in its whole	Body dysphoria
	Am I…? Yes, you are: validation from partners, colleagues and social media	Belonging Acceptance Relief

## Data Availability

Data is contained within the article or Supplementary Materials.

## Supplementary Materials

The following supporting information can be downloaded at: https://www.mdpi.com/article/10.3390/healthcare13121467/s1.
