# Supplementary material for: The Emotional Universe of Nonbinary Parents: A Hermeneutic Study
Source: Healthcare (Basel). 2025 Jun 18;13(12):1467. doi: 10.3390/healthcare13121467 (PMC12193258; doi:10.3390/healthcare13121467)
Supplement: Supplementary file 1 [file healthcare-13-01467-s001.zip › healthcare-3657919-supplementary.pdf]

Supplementary material.

Overview of Bisquerra’s Taxonomy of Emotions

1. Origin and Purpose

Bisquerra’s taxonomy of emotions (2014, 2018, 2022) was developed to provide a comprehensive and pedagogically accessible framework for understanding the complexity of human affective experience. It is grounded in emotional education and hermeneutic phenomenology, aiming to foster emotional awareness, empathy, and ethical sensitivity. Compared to other widely used models, such as Plutchik’s Wheel of Emotions or Ekman’s six basic emotions, Bisquerra’s taxonomy offers several advantages for qualitative and interpretive research. It provides high emotional granularity by including nuanced, social, and ambiguous emotions; it aligns closely with hermeneutic approaches due to its metaphorical structure (see below); and supports a culturally resonant and interpretively rich analysis of affective discourse.

2. Structure of the Taxonomy

The taxonomy organizes emotions into five broad categories:

- Positive Emotions: Emotions that promote well-being and flourishing (e.g., joy, love, gratitude).

- Negative Emotions: Emotions that signal discomfort or threat (e.g., sadness, anger, fear).

- Ambiguous Emotions: Emotions that may be experienced as either pleasant or unpleasant depending on context (e.g., surprise, curiosity, confusion).

- Primary Emotions: Basic, universal emotions often considered innate (e.g., disgust).

- Social Emotions: Emotions that arise in interpersonal contexts and are shaped by social norms (e.g., guilt, pride).

Each category includes a range of nuanced emotional states, allowing for greater emotional granularity and contextual interpretation.

3. Metaphorical and Hermeneutic Foundation

Bisquerra’s taxonomy is a classification system and a conceptual framework grounded in metaphor and hermeneutics. It presents the emotional world as a “Universe of Emotions,” a metaphor that invites us to explore affective life as a vast, dynamic, and

interconnected space, much like the cosmos. This metaphorical framing serves several purposes:

- It emphasizes the complexity and expansiveness of emotional experience, suggesting that emotions are not isolated or static but somewhat fluid, relational, and evolving.
- It supports a hermeneutic approach, where emotions are interpreted within the context of individuals' lifeworlds, histories, and social realities. This aligns with interpretive phenomenology, which views emotions as meaning-making forces that shape and are shaped by experience.
- It integrates poetic and symbolic elements by drawing on metaphors and imagery that evoke emotional resonance and ethical reflection. For instance, the taxonomy is framed as a "Universe of Emotions," inviting readers to imagine emotions as celestial bodies—vast, interconnected, and in constant motion. This metaphor is enriched by references to Rumi's poem The Guest House, which portrays emotions as visitors to be welcomed and understood, and to Florence Nightingale's "Lady's Lamp," symbolizing empathy as a guiding light in the care of others. These poetic references are not merely decorative; they serve as interpretive tools that deepen the understanding of the emotional experience and support a more humanistic and compassionate approach to affective care.

In this framework, the Universe of Emotions becomes a guest house, and empathy is the lamp that illuminates each of its lodgings, revealing, embracing, and caring for the full spectrum of human affective experience.

4. Visual representation

The visual representations in this section (Figures S1-4) synthesize the three hermeneutic foundations that structure our methodological approach: the Universe of Emotions, the Guest House, and the Lady's Lamp. Rather than functioning as a mere illustration, this figure embodies the study's interpretive logic and ethical orientation.

The Universe of Emotions (Figure S1), as conceptualized by Bisquerra, provides the structural and semantic foundation for classifying affective experiences. Its metaphorical framing as a cosmos of emotional galaxies allows for a dynamic and interconnected understanding of emotions, aligning with the hermeneutic emphasis on fluidity, context, and meaning-making.

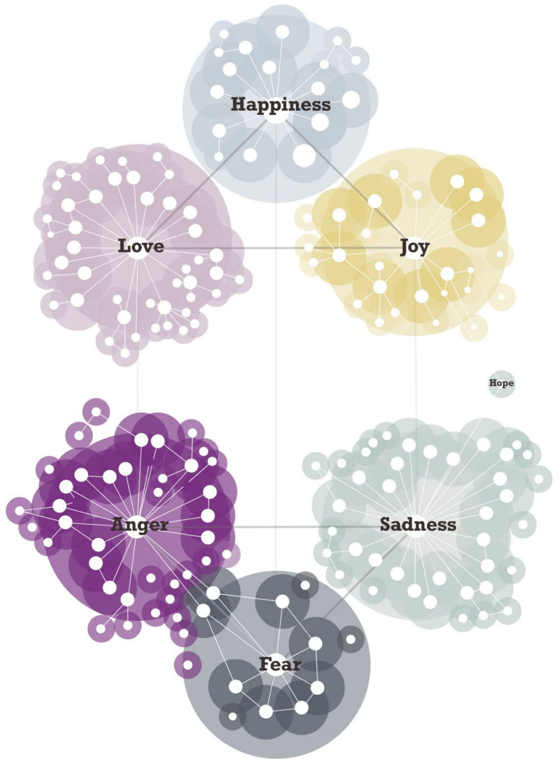

Figure S1. Main galaxies in Universe of Emotions

The Guest House (Figure S2), inspired by Rumi's poem, introduces a phenomenological and ethical lens through which emotions are interpreted. Emotions are not treated as fixed states but as visitors—some welcome, others disruptive—each carrying a message. This metaphor supports the interpretive openness and emotional hospitality that guide our analysis and resonates with the idea of the self as a space of encounter and transformation.

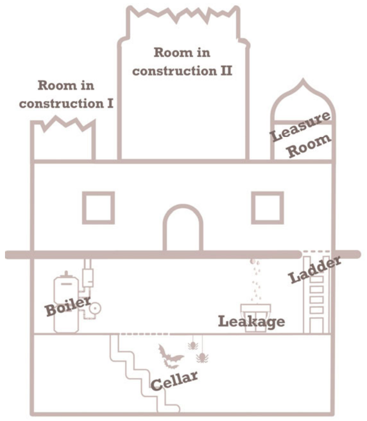

Figure S2. The Ghest House and its diverse lodgings

The Lady's Lamp (Figure S3), drawing on the symbolic figure of Florence Nightingale, represents empathy as both a moral disposition and a methodological tool. It is the light that allows us to navigate the emotional architecture of the Guest House, illuminating each of its lodgings with care and attentiveness. Empathy, in this sense, is a guiding principle and a condition for the double hermeneutic process that characterizes qualitative inquiry.

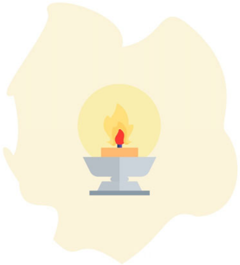

Figure S3. Empathy: The Lady's Lamp

These three elements form a coherent visual and conceptual framework supporting the interpretive process (Figure S4). The figure integrates metaphor, taxonomy, and ethical orientation into a unified model that reflects the epistemological commitments of the study. It invites the reader to imagine the emotional world not as a static map but as a living, inhabited space that must be explored with light, welcomed with openness, and interpreted with care.

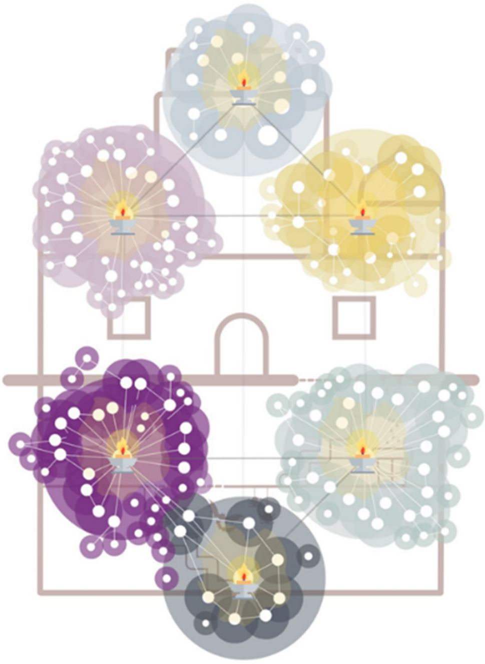

Figure S4. Explanotory framework of the methodological proposal

5. Use in International Research

Although initially developed within the Spanish-speaking academic context, Bisquerra's taxonomy has proven to be a valuable tool in international research, as evidenced by its application in peer-reviewed English-language publications such as Magro-Morillo et al. (2020) in Intensive and Critical Care Nursing. In addition to this study, other scholarly works have also employed the taxonomy in similar lines of inquiry, highlighting its versatility and relevance across diverse cultural and clinical settings, particularly in hermeneutic and affective research.
